# Oculogyric Crisis During Chronic Aripiprazole Therapy: A Diagnostic Challenge in the Emergency Department

**DOI:** 10.1002/ccr3.72067

**Published:** 2026-02-16

**Authors:** Kohei Tokioka, Tsuyoshi Nojima, Ippei Matsuo, Kohei Tsukahara, Atsunori Nakao

**Affiliations:** ^1^ Department of Emergency, Critical Care, and Disaster Medicine, Faculty of Medicine, Dentistry, and Pharmaceutical Sciences Okayama University Japan

**Keywords:** acute dystonia, aripiprazole, drug‐induced movement disorder, oculogyric crisis

## Abstract

Oculogyric crisis can occur even during chronic, stable aripiprazole therapy without recent dose escalation. In patients with acute upward eye deviation and an otherwise normal neurologic examination, medication review is key to recognizing drug‐induced dystonia and avoiding unnecessary neurologic workup.

A 21‐year‐old male with depression had been taking aripiprazole 24 mg daily for an extended period; there had been no recent dose escalation, overdose, or adherence changes prior to symptom onset. He was not taking any other medications known to be associated with dystonia. While at work, a colleague noticed an upward deviation of his eyes. He visited an ophthalmologist, but no abnormalities were found. Later that day, he developed difficulty maintaining forward gaze and presented to the emergency department. He was alert and oriented, with stable vital signs. Neurological examination was normal except for sustained upward ocular deviation (Figure 1). Laboratory tests and neuroimaging revealed no abnormalities. Seizure and acute cerebrovascular disease were excluded based on normal neurologic examination and imaging. Accordingly, a diagnosis of aripiprazole‐induced oculogyric crisis (OGC), representing an acute dystonic reaction, was made. As the symptoms were mild and improving, conservative management without anticholinergic therapy was chosen. The evening dose of aripiprazole was withheld, and the dosage was later reduced to 12 mg, leading to full recovery within three days.

**FIGURE 1:**
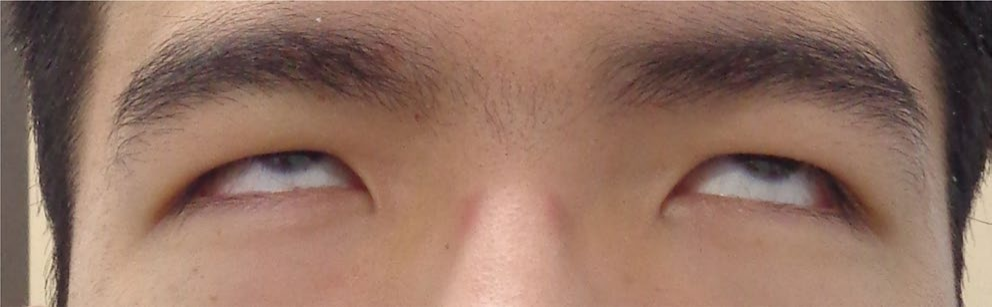
Oculogyric crisis during emergency department evaluation. Clinical photograph demonstrating sustained upward deviation of both eyes with preserved consciousness in a patient receiving chronic aripiprazole therapy.

This case underscores the importance of recognizing drug‐induced dystonic reactions that may mimic acute neurologic emergencies. OGC is a rare form of focal dystonia characterized by sustained, bilateral upward eye deviation, resulting from involuntary contraction of the extraocular muscles [1].

The pathophysiology of neuroleptic‐induced acute dystonia is thought to involve relative dopaminergic blockade or functional D2 receptor antagonism, leading to an imbalance between dopaminergic and cholinergic activity within the basal ganglia [1]. Aripiprazole, an atypical second‐generation antipsychotic, acts as a partial agonist at dopamine D2 and serotonin 5‐HT1A receptors and an antagonist at 5‐HT2A receptors with a low intrinsic risk of extrapyramidal side effects [2]. Nevertheless, dystonic reactions may still occur, particularly in individuals with certain predisposing factors such as young age, male sex, concurrent use of psychoactive substances like cocaine, and a history of acute dystonia [2, 3]. The prevalence of oculogyric crisis associated with antipsychotic medication has been reported to range between 0.9% and 3.4% [1].

OGC can be misinterpreted as seizure, encephalitis, or psychogenic disorder, often leading to unnecessary neuroimaging or anticonvulsant administration. Unlike most previously reported cases, this patient developed oculogyric crisis during chronic aripiprazole therapy without recent dose escalation or overdose [3].

Although anticholinergic agents such as biperiden are generally considered first‐line therapy for drug‐induced acute dystonia, the symptoms in this case resolved spontaneously without pharmacologic intervention. The spontaneous improvement may have resulted from restoration of dopaminergic–cholinergic balance as the steady‐state serum concentration of aripiprazole was reestablished [1, 2]. Similar spontaneous remissions have occasionally been reported in mild cases of oculogyric crisis once the offending agent was metabolized [2].

In patients presenting with acute‐onset abnormal eye movements or dystonic posturing, obtaining a thorough medication history is essential for diagnosis. When neurological examination and laboratory investigations reveal no abnormalities, clinicians should consider drug‐induced dystonia, particularly in individuals who have ingested dopamine receptor‐modulating agents such as aripiprazole. Early recognition of this condition can prevent misdiagnosis, avoid unnecessary interventions, and ensure prompt, effective management.

## Author Contributions

**Kohei Tokioka:** conceptualization, investigation, project administration, writing – original draft. **Tsuyoshi Nojima:** writing – review and editing. **Ippei Matsuo:** writing – review and editing. **Kohei Tsukahara:** writing – review and editing. **Atsunori Nakao:** supervision, writing – review and editing.

## Funding

The authors have nothing to report.

## Ethics Statement

The authors have nothing to report.

## Consent

Written informed consent was obtained from the patient for the publication of this case report including the images.

## Conflicts of Interest

The authors declare no conflicts of interest.

## Data Availability

Data sharing is not applicable to this article as no datasets were generated or analyzed during the current study.
